# Identification of ANC-3 as a Novel Therapeutic Candidate for Anaplastic Thyroid Cancer Through Drug Screening and Multi-Platform Validation

**DOI:** 10.3390/ijms27125222

**Published:** 2026-06-09

**Authors:** Dorjsuren Tsagaankhuu, Hyunwoo Baek, Jungyoon Choi, Soonyoung Kwon

**Affiliations:** 1 Department of Otorhinolaryngology–Head and Neck Surgery, Korea University Ansan Hospital, Korea University College of Medicine, Ansan 15355, Republic of Korea; dorjsuren9999@gmail.com; 2 Biomedical Research Center, Korea University Ansan Hospital, Korea University College of Medicine, Ansan 15355, Republic of Korea; bhw0810@korea.ac.kr; 3 Division of Oncology and Hematology, Department of Internal Medicine, Korea University Ansan Hospital, Korea University College of Medicine, Ansan 15355, Republic of Korea; jungyoonchoi@korea.ac.kr

**Keywords:** anaplastic thyroid carcinoma, zebrafish xenograft, drug screening, aridine-3,6-diamine hydrochloride

## Abstract

Anaplastic thyroid carcinoma (ATC) is a rare but highly aggressive malignancy characterized by rapid progression, early metastasis, and extremely poor survival outcomes. Effective therapeutic options remain limited, highlighting the need for efficient and biologically relevant preclinical drug-discovery platforms. In this study, high-throughput compound screening using human ATC cell lines identified ANC-3 as a potential anticancer candidate. Its antitumor activity was evaluated through cytotoxicity and functional assays, zebrafish xenograft validation with live fluorescence imaging, colony-formation assays, and bulk RNA sequencing with pathway enrichment analyses. ANC-3 demonstrated consistent antitumor effects by significantly inhibiting cell viability, migration, invasion, and clonogenic survival, while also suppressing tumor growth in zebrafish xenograft models. Transcriptomic analyses revealed modulation of multiple oncogenic pathways, including MAPK, Ras, and NF-κB signaling. Collectively, these findings support zebrafish xenograft-based screening as a rapid and scalable platform for ATC drug discovery and suggest ANC-3 as a promising multi-pathway inhibitor warranting further preclinical development.

## 1. Introduction

Anaplastic thyroid carcinoma (ATC) is a rare but nearly universally fatal malignancy originating from thyroid follicular cells and represents the most aggressive form of thyroid cancer [1]. Although ATC accounts for only 1–2% of thyroid carcinomas, it contributes disproportionately to thyroid cancer-related mortality, with a median overall survival of approximately 5–6 months after diagnosis [2,3]. The American Joint Committee on Cancer classifies all ATCs as stage IV disease regardless of tumor size or metastatic status, reflecting its intrinsically aggressive biology [4]. Clinically, ATC is characterized by rapid local invasion, early distant metastasis, and resistance to conventional therapies, and its prognosis has remained largely unchanged over recent decades despite advances in differentiated thyroid carcinoma management [5,6].

Molecular evidence suggests that many ATCs arise through dedifferentiation of pre-existing differentiated thyroid carcinoma via accumulation of additional genetic alterations [7,8]. Genomic studies demonstrate retention of early driver mutations such as BRAF or RAS, accompanied by high-risk alterations including TP53, TERT promoter mutations, and dysregulation of the PI3K–AKT–mTOR pathway, which together contribute to genomic instability and aggressive tumor behavior [9,10,11]. Current treatment strategies rely on multimodal approaches, including surgery, radiotherapy, and systemic therapy when feasible; however, survival benefits remain modest [12]. Although BRAF/MEK-targeted therapy has improved outcomes in BRAFV600E-mutated ATC, its applicability is limited to a molecular subset of patients, and resistance frequently develops [7,11].

Given the fulminant clinical course and limited therapeutic efficacy, there is a critical need for efficient preclinical platforms capable of identifying novel therapeutic agents and rational drug combinations. Conventional in vitro systems fail to recapitulate tumor heterogeneity and microenvironmental complexity, whereas mammalian in vivo models are resource-intensive and poorly suited for rapid screening [13,14]. In this context, zebrafish xenograft models have emerged as a promising intermediate platform that enables rapid tumor engraftment, real-time visualization of tumor behavior, and high-throughput drug evaluation [15,16,17]. These characteristics make zebrafish systems particularly attractive for early-stage therapeutic prioritization in highly aggressive cancers such as ATC [10,18].

In this study, we aimed to identify novel anticancer candidates through high-efficiency drug screening using the ATC-derived SNU80 cell line and to validate the antitumor efficacy of the selected compound, ANC-3, using integrative in vitro functional assays, a zebrafish xenograft model, and transcriptomic analyses. We further sought to elucidate the molecular mechanisms underlying its anticancer activity by examining global gene-expression changes and pathway-level modulation associated with ATC progression.

## 2. Results

### 2.1. Identification of ANC-3 Through Drug Screening in ATC Cells

**Figure 1:**
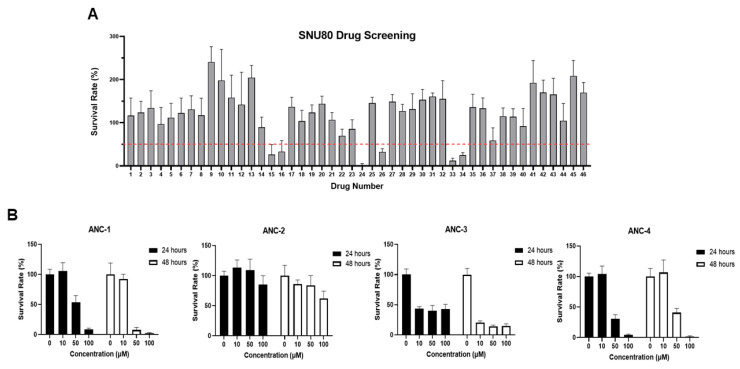
Drug screening and dose-dependent effects of candidate compounds in SNU80 cells. (**A**) Screening of 46 selected compounds in SNU80 cells. Cell survival rates were normalized to the untreated control (100%). The red dashed line indicates the 50% survival threshold. Six compounds reduced cell viability to below 50%. (**B**) Dose- and time-dependent effects of selected candidate compounds (ANC-1, ANC-2, ANC-3, and ANC-4) on SNU80 cell viability. Cells were treated with the indicated concentrations (0–100 μM) for 24 and 48 h. Cell survival was expressed relative to untreated controls. Data are presented as mean ± SD.

To identify effective anticancer candidates, compounds potentially associated with cancer-related signaling pathways were first selected based on drug annotation data from the MedChemExpress (MCE) compound library, and a total of 46 compounds were chosen for screening in SNU80 cells using a Cell Counting Kit-8 (CCK-8)-based cell viability assay. Their effects on cell viability were evaluated (Figure 1A), and six compounds were found to significantly reduce cell viability (Table 1). Among these, two compounds (No. 15 and No. 26), which had been previously reported to possess anticancer activity, were excluded to prioritize novel candidates. The remaining four compounds were designated ANC-1 to ANC-4 [19,20,21].

Subsequent dose- and time-dependent analyses were performed to further assess the anticancer effects of these candidates. Three compounds, excluding ANC-2, significantly reduced SNU80 cell viability, with ANC-3 exhibiting the strongest inhibitory effect among the selected candidates (Figure 1B). These findings suggest that a mechanism-guided selection strategy based on the MCE library can be effective for identifying novel anticancer compounds, and support ANC-3 as a promising lead candidate for further functional and mechanistic investigations.

### 2.2. Toxicity Evaluation of ANC Compounds in Zebrafish Larvae

**Figure 2:**
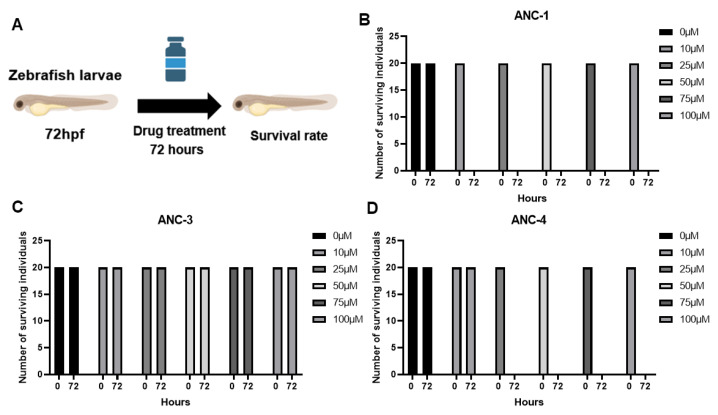
Toxicity evaluation of ANC compounds in zebrafish larvae. (**A**) Experimental design for toxicity assessment in zebrafish larvae treated with ANC compounds at different concentrations for 72 h. (**B**–**D**) Survival rates of zebrafish larvae following treatment with ANC-1, ANC-3, and ANC-4, respectively. No significant differences in survival were observed across treatment groups, indicating low toxicity. Data are presented as mean ± SD (n = 20 per group).

To assess in vivo toxicity, zebrafish larvae at 72 h post-fertilization (hpf) were treated with increasing concentrations of ANC compounds for 72 h (Figure 2A). Survival analysis showed that ANC-1, ANC-3, and ANC-4 did not significantly affect zebrafish viability across tested concentrations, indicating low toxicity (Figure 2B–D). These results suggest that ANC-3 is a safe candidate for further in vivo evaluation.

### 2.3. ANC-3 Suppresses Tumor Growth in Zebrafish Xenograft Model

**Figure 3:**
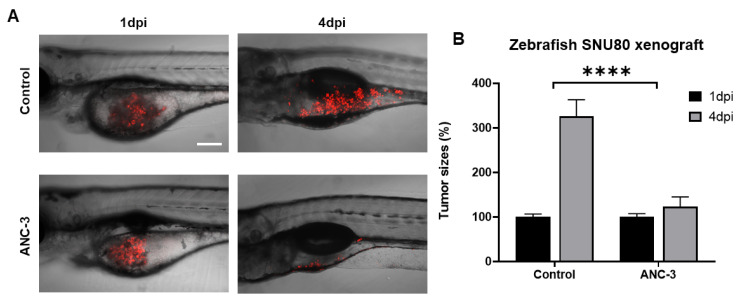
ANC-3 suppresses tumor growth in a zebrafish xenograft model. (**A**) Representative fluorescence images of zebrafish xenografts injected with Deep Red-labeled SNU80 cells at 1 dpi and 4 dpi, with or without ANC-3 treatment. (**B**) Quantification of tumor burden based on fluorescence intensity. ANC-3 treatment significantly reduced tumor growth compared to the control group. Data are presented as mean ± SD (n = 14 per group, **** *p* < 0.0001).

To investigate the antitumor effect of ANC-3 in vivo, a zebrafish xenograft model was established. Fluorescently labeled SNU80 cells were injected into zebrafish embryos, and tumor growth was monitored at 1 day post-injection (dpi) and 4 dpi (Figure 3A). ANC-3 treatment significantly reduced tumor fluorescence intensity compared to the control group. Quantitative analysis confirmed a marked decrease in tumor size in ANC-3-treated embryos (Figure 3B).

### 2.4. ANC-3 Inhibits Migration and Invasion of ATC Cells

**Figure 4:**
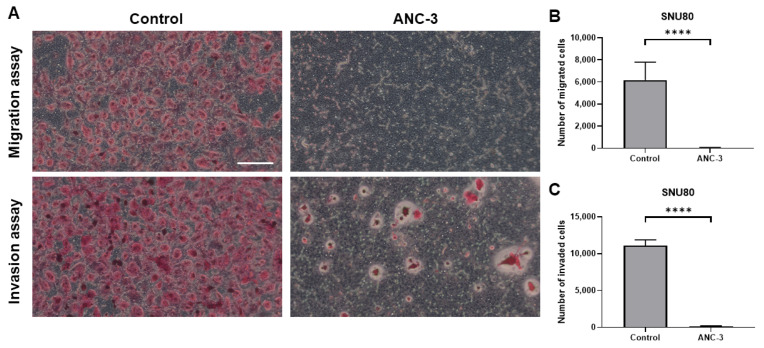
ANC-3 inhibits migration and invasion of SNU80 cells. (**A**) Representative images of migrated and invaded SNU80 cells following treatment with ANC-3 using Transwell assays. Migration assays were performed with cells seeded in serum-free medium in the upper chamber, whereas invasion assays were conducted using Matrigel-coated inserts. Cells that migrated or invaded through the membrane were visualized by hematoxylin and eosin (H&E) staining. Scale bar = 100 μm. (**B**) Quantification of migrated cells per well. (**C**) Quantification of invaded cells per well. ANC-3 treatment significantly reduced both migratory and invasive capacities of SNU80 cells compared with the control group. Data are presented as mean ± SD. (n = 9 per group, **** *p* < 0.0001).

The effect of ANC-3 on cell motility was evaluated using migration and invasion assays. ANC-3 treatment markedly reduced the number of migrated and invaded SNU80 cells compared to control conditions (Figure 4A). Quantitative analysis confirmed a significant decrease in both migration and invasion abilities following ANC-3 treatment (Figure 4B,C).

### 2.5. ANC-3 Suppresses Clonogenic Capacity of SNU80 Cells

**Figure 5:**
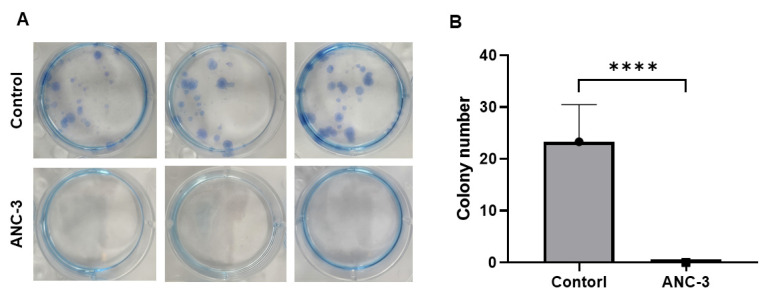
ANC-3 suppresses clonogenic survival and long-term proliferative capacity of SNU80 cells. (**A**) Representative images of colony formation assays showing a marked reduction in both colony number and colony density in SNU80 cells following treatment with ANC-3 compared with untreated control cells. (**B**) Quantitative analysis of colony numbers demonstrating that ANC-3 treatment significantly inhibited clonogenic survival and reduced the colony-forming ability of SNU80 cells, indicating impaired long-term proliferative potential. These findings suggest that ANC-3 effectively suppresses sustained tumor cell growth and reproductive viability in ATC cells. Data are presented as mean ± SD. (n = 6 per group, **** *p* < 0.0001).

Colony formation assays were conducted to assess the long-term proliferative and clonogenic potential of anaplastic thyroid cancer cells following ANC-3 treatment. Compared with untreated control cells, ANC-3-treated cells exhibited a marked reduction in both the number and size of colonies formed, indicating substantial impairment of long-term survival and reproductive capacity (Figure 5A). In addition to suppressing colony initiation, ANC-3 treatment significantly limited sustained clonal expansion, resulting in fewer surviving colonies with reduced growth potential. Quantitative analysis confirmed a significant decrease in colony numbers in the ANC-3-treated group relative to controls, demonstrating robust inhibition of clonogenic survival (Figure 5B). These findings suggest that ANC-3 effectively suppresses the long-term proliferative potential of ATC cells and may impair tumor regrowth capacity.

### 2.6. Transcriptomic Profiling Reveals Molecular Mechanisms of ANC-3

**Figure 6:**
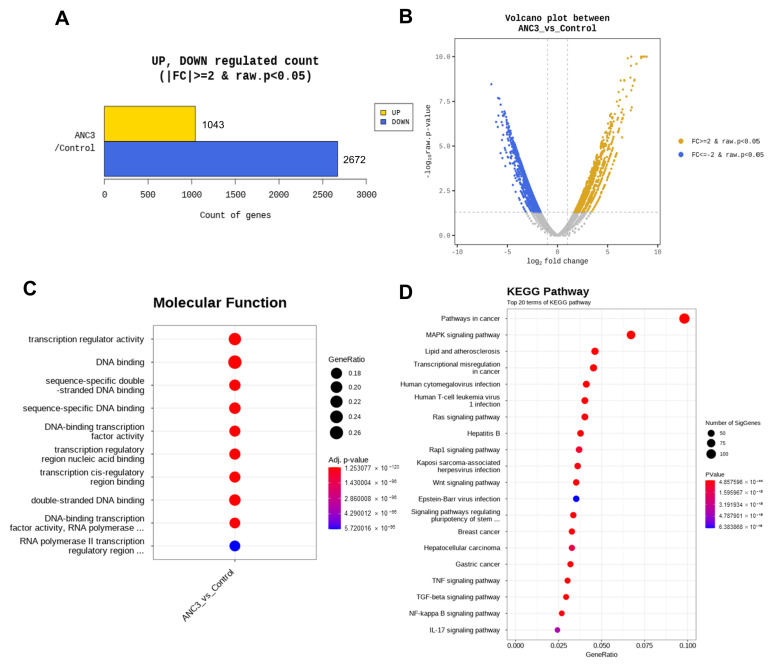
Transcriptomic Profiling Reveals Molecular Mechanisms of ANC-3. To elucidate the molecular mechanisms underlying the effects of ANC-3, RNA sequencing was performed. Differential expression analysis identified a substantial number of upregulated and downregulated genes (**A**,**B**). Gene Ontology analysis revealed enrichment in molecular functions associated with transcription regulation and DNA binding (**C**). KEGG pathway analysis further indicated that ANC-3 modulates multiple cancer-related signaling pathways (**D**), suggesting a multi-target mechanism of action.

To elucidate the molecular mechanisms underlying the antitumor effects of ANC-3, bulk RNA sequencing was performed in ANC-3-treated SNU80 cells compared with untreated controls. Differential gene expression analysis identified substantial transcriptomic alterations, including 1483 upregulated and 2672 downregulated genes (|FC| > 2, adjusted *p* < 0.05) (Figure 6A), which were further visualized by volcano plot analysis (Figure 6B). Gene Ontology analysis of molecular function revealed significant enrichment of genes associated with transcription regulator activity, DNA binding, sequence-specific double-stranded DNA binding, transcription factor activity, and RNA polymerase II regulatory region binding, suggesting that ANC-3 broadly affects transcriptional regulatory programs (Figure 6C). KEGG pathway enrichment analysis further demonstrated significant modulation of multiple cancer-related signaling pathways, including MAPK, Ras, TNF, NF-κB, and IL-17 signaling pathways, as well as pathways associated with viral infection, apoptosis-related signaling, and hepatocellular carcinoma (Figure 6D). Collectively, these findings suggest that ANC-3 exerts its antitumor activity through multi-target modulation of diverse oncogenic and inflammatory signaling networks.

## 3. Discussion

ATC is one of the most aggressive and lethal malignancies, characterized by rapid progression, high metastatic potential, and resistance to conventional therapies [11,22,23]. Despite advances in targeted therapies, effective treatment options for ATC remain limited, underscoring the urgent need for novel therapeutic agents [11,24,25].

In this study, we performed a targeted drug screening approach and identified ANC-3 as a potent inhibitor of ATC cell viability. Among 46 selected compounds enriched for cancer-related signaling pathways, ANC-3 exhibited the most pronounced cytotoxic effect in SNU80 cells. Importantly, its inhibitory activity was consistently observed in a dose-dependent manner, supporting its potential as a pharmacologically active candidate. Notably, Acridine-3,6-diamine hydrochloride-related compounds have previously been investigated in drug delivery and imaging applications, highlighting their broader biomedical relevance and potential translational applicability [21].

A critical consideration in early-stage drug development is toxicity. Using a zebrafish model, we demonstrated that ANC-3 did not significantly affect survival rates across tested concentrations, indicating a favorable safety profile in vivo. This finding is particularly important, as many compounds with strong in vitro efficacy fail to progress due to systemic toxicity.

Beyond cytotoxicity, metastatic behavior is a key determinant of ATC prognosis. Our results showed that ANC-3 significantly suppressed both migration and invasion of SNU80 cells, suggesting that it may interfere with mechanisms underlying tumor dissemination. In addition, ANC-3 markedly reduced clonogenic survival, indicating its ability to impair long-term proliferative and tumorigenic potential.

To further validate its antitumor efficacy in vivo, we employed a zebrafish xenograft model. ANC-3 treatment led to a significant reduction in tumor burden, as evidenced by decreased fluorescence intensity. This result demonstrates that the anticancer effects of ANC-3 are not limited to in vitro conditions but are also effective in a living organism, supporting its translational potential.

At the molecular level, RNA sequencing and subsequent pathway analyses revealed that ANC-3 modulates multiple cancer-related signaling pathways. Gene Ontology analysis indicated enrichment in transcriptional regulation and DNA-binding functions, while KEGG pathway analysis highlighted alterations in key oncogenic pathways. These findings suggest that ANC-3 exerts its antitumor effects through a multi-target mechanism rather than a single pathway inhibition, which may be advantageous in overcoming resistance mechanisms commonly observed in ATC [26,27].

Despite these promising findings, several limitations should be acknowledged. First, this study was primarily conducted using a single ATC cell line, which may not fully represent the heterogeneity of ATC. Second, although the zebrafish model provides rapid and efficient in vivo validation, it does not fully recapitulate the complexity of mammalian tumor microenvironments. In addition, comprehensive pharmacokinetic and toxicological evaluations, including ADME (Absorption, Distribution, Metabolism, and Excretion) analysis of ANC-3, were not performed in the current study and will be necessary to further assess its clinical translational potential. Therefore, further studies using additional ATC models, including patient-derived cells and murine xenograft systems, are warranted.

## 4. Materials and Methods

### 4.1. Cell Lines and Culture Conditions

The human ATC cell line SNU80 was obtained from a certified cell repository and maintained under standardized in vitro culture conditions routinely applied for aggressive thyroid cancer models. Cells were cultured in RPMI-1640 medium supplemented with 10% fetal bovine serum (FBS) and 1% penicillin–streptomycin to prevent microbial contamination and ensure optimal proliferation [19]. Cultures were incubated at 37 °C in a humidified atmosphere containing 5% CO_2_, representing conventional physiological conditions for mammalian cell growth and metabolic stability.

### 4.2. Drug Screening and Cell Viability Assay

A high-throughput drug screening assay was performed using a commercially available MedChemExpress (MCE) compound library to identify potential anticancer candidates for ATC. From a total of 1165 small-molecule compounds, 46 agents targeting cancer-associated signaling pathways were pre-selected based on curated databases and prior experimental evidence. SNU80 cells were seeded into 96-well plates at a density of 2 × 10^4^ cells per well and treated with each compound for 48 h. Cell viability was measured using the CCK-8 assay, and absorbance was recorded at 450 nm after 2 h of incubation using a SpectraMax Plus 384 microplate reader (Molecular Devices, San Jose, CA, USA), according to the manufacturer’s protocol.

For further evaluation, selected compounds were subjected to dose- and time-dependent analyses. Cells were treated with 0, 10, 50, and 100 μM of each compound for 24 and 48 h.

### 4.3. Migration and Invasion Assays

The impact of ANC-3 on the migratory and invasive potential of SNU80 cells was investigated using a Transwell chamber system with 8-μm pore size inserts (SPL Life Sciences, Pocheon, Republic of Korea), an established method for assessing tumor cell motility and extracellular matrix penetration. For migration assays, 2 × 10^4^ cells suspended in serum-free medium were seeded into the upper chamber, while complete medium was placed in the lower compartment as a chemoattractant.

For invasion assays, identical conditions were applied except that the inserts were pre-coated with Matrigel, thereby mimicking basement membrane architecture and enabling assessment of invasive capacity. ANC-3 was administered immediately after cell seeding, and cells were incubated for 72 h, a time frame sufficient to observe significant motility changes in aggressive carcinoma phenotypes.

Following incubation, non-migrated cells were removed, and cells traversing the membrane were fixed with 4% paraformaldehyde and stained using hematoxylin and eosin (H&E) for morphological visualization. Quantification was performed by counting stained cells in randomly selected microscopic fields, a standard semi-quantitative approach for invasion assays.

### 4.4. Colony Formation Assay

A colony formation assay was conducted to evaluate the long-term proliferative and clonogenic capacity of SNU80 cells after ANC-3 exposure, which reflects sustained tumorigenic potential beyond short-term cytotoxicity. Cells (2 × 10^3^ per well) were plated into 6-well plates and treated with ANC-3 for approximately 10 days, allowing sufficient time for colony development.

Colonies were subsequently fixed using 100% methanol and stained with 4% methylene blue, which enhances visualization of viable cell clusters. Macroscopically distinguishable colonies containing more than 50 cells were counted manually under light microscopy to obtain quantitative measurements of clonogenic survival.

### 4.5. Zebrafish Maintenance and Embryo Handling

Zebrafish were maintained, handled, and bred in strict accordance with the guidelines approved by the Institutional Animal Care and Use Committee (IACUC) of Korea University. Adult fish were housed at 26–28 °C under a controlled 14 h light/10 h dark cycle [20]. Embryos were obtained by natural spawning in breeding tanks containing one male and two female zebrafish. Following collection, embryos were maintained at 28.5 °C in E3 medium supplemented with 0.2 mM phenylthiourea (PTU) for 48 h to inhibit melanization [21]. Wild-type zebrafish were obtained from the Korea University Zebrafish Translational Medical Research Center.

### 4.6. Zebrafish Xenograft Model

A zebrafish xenograft model was established to evaluate in vivo antitumor efficacy, a system increasingly recognized for rapid, cost-efficient, and high-throughput oncology drug validation. SNU80 cells were labeled with CellTracker fluorescent dye and microinjected into the yolk of 48 hpf zebrafish embryos using a WPI microinjection system (World Precision Instruments, Sarasota, FL, USA), a developmental stage that permits efficient engraftment without adaptive immune rejection [15].

Following transplantation, embryos were treated with ANC-3, and fluorescent images were captured at 1 dpi and 4 dpi using a LSM 900 confocal microscope (Carl Zeiss AG, Oberkochen, Germany).Tumor burden was quantified by measuring fluorescent area with ImageJ software version 1.53V, and tumor progression was calculated as the relative change in fluorescence intensity between time points. This approach enables objective quantification of xenograft growth dynamics and therapeutic response.

### 4.7. RNA Sequencing and KEGG Pathway Analysis

Total RNA was extracted from SNU80 cells treated with ANC-3 for 24 h and from untreated control cells using validated isolation protocols to preserve RNA integrity. RNA quality and concentration were assessed prior to library preparation. Bulk RNA sequencing (RNA-seq) was performed to comprehensively evaluate genome-wide transcriptional alterations. Differentially expressed genes (DEGs) were identified using thresholds of an absolute fold change ≥ 2 and a raw *p*-value < 0.05.

To determine the biological relevance of the identified DEGs, Kyoto Encyclopedia of Genes and Genomes (KEGG) pathway enrichment analysis was conducted. The significantly upregulated and downregulated genes were mapped to the KEGG database to identify enriched signaling pathways and functional networks. Pathways with a *p*-value < 0.05 were considered statistically significant. This analysis enabled the identification of key cancer-related pathways potentially associated with the molecular effects of ANC-3 treatment.

### 4.8. Statistical Analysis

All experiments were independently repeated at least three times to ensure reproducibility and statistical reliability. Quantitative data were expressed as mean ± standard deviation (SD). Comparisons between two experimental groups were performed using the Student’s *t*-test, and *p* < 0.05 was considered statistically significant. Statistical analyses were conducted using GraphPad Prism software version 9.0.0V, a commonly accepted platform in biomedical data analysis.

## 5. Conclusions

ATC remains a highly aggressive malignancy with limited effective therapeutic options. In this study, ANC-3 was identified through systematic drug screening and demonstrated significant antitumor activity across in vitro and in vivo models. ANC-3 inhibited cellular proliferation, migration, invasion, and clonogenic survival of ATC cells, and effectively suppressed tumor growth in a zebrafish xenograft system.

Transcriptomic analyses further revealed that its anticancer activity is associated with broad transcriptional reprogramming and coordinated modulation of multiple oncogenic signaling pathways. Collectively, these findings position ANC-3 as a promising investigational candidate and support zebrafish-based drug-screening platforms as efficient translational tools for accelerating therapeutic discovery in ATC.

## Figures and Tables

**Table 1:** Selected candidate compounds identified from drug screening. Summary of compounds that reduced SNU80 cell viability in the primary screening. Four compounds were designated as ANC-1 to ANC-4 and selected for subsequent experiments.

Drug Number	Labeling of Drug	Drug Name
15	-	6-Bromo-4-hydroxyquinazoline
16	ANC-1	4,4′-(9H-Fluorene-9,9-diyl)diphenol
24	ANC-2	N-(P-Tolyl)cinnamamide
26	-	1,3-Bis(4-aminophenoxy)benzene
33	ANC-3	Acridine-3,6-diamine hydrochloride
34	ANC-4	N1-Isopropyl-N4-phenylbenzene-1,4-diamine

## Data Availability

Data available upon request.

## Abbreviations

The following abbreviations are used in this manuscript:

ATC	Anaplastic Thyroid Cancer
CCK-8	Cell Counting Kit-8
FBS	Fetal Bovine Serum
MCE	MedChemExpress
hpf	Hours Post Fertilization
dpi	Days Post Injection
DEGs	Differentially Expressed Genes
GO	Gene Ontology
KEGG	Kyoto Encyclopedia of Genes and Genomes
SD	Standard Deviation

## References

[1] Lawless A.K., Kumar S., Bindra J., Sywak M., Chou A., Turchini J., Papachristos A., Wijewardene A., Sidhu S., Ahadi M. (2024). Anaplastic thyroid cancer: A review of recent evidence and summary of an Australian institutional protocol. Asia Pac. J. Clin. Oncol..

[2] Guo H., Zhang J., Jia Y., Liu Z., Qi Y., Sun C., Cai Z., Wu J. (2025). Trends in incidence, mortality, and conditional survival of anaplastic thyroid cancer over the last two decades in the USA. Front. Endocrinol..

[3] Lamartina L., Jannin A., Decaussin-Petrucci M., Bardet S., Escande A., Ciappuccini R., Borson Chazot F., Al Ghuzlan A., Do Cao C., Hadoux J. (2025). ENDOCAN TUTHYREF network consensus recommendations: Anaplastic thyroid cancer. Ann. Endocrinol..

[4] Bible K.C., Kebebew E., Brierley J., Brito J.P., Cabanillas M.E., Clark T.J., Di Cristofano A., Foote R., Giordano T., Kasperbauer J. (2021). 2021 American Thyroid Association Guidelines for Management of Patients with Anaplastic Thyroid Cancer: American Thyroid Association Anaplastic Thyroid Cancer Guidelines Task Force. Thyroid.

[5] Molinaro E., Romei C., Biagini A., Sabini E., Agate L., Mazzeo S., Materazzi G., Sellari-Franceschini S., Ribechini A., Torregrossa L. (2017). Anaplastic thyroid carcinoma: From clinicopathology to genetics and advanced therapies. Nat. Rev. Endocrinol..

[6] Landa I., Ibrahimpasic T., Boucai L., Sinha R., Knauf J.A., Shah R.H., Dogan S., Ricarte-Filho J.C., Krishnamoorthy G.P., Xu B. (2016). Genomic and transcriptomic hallmarks of poorly differentiated and anaplastic thyroid cancers. J. Clin. Investig..

[7] Pozdeyev N., Gay L.M., Sokol E.S., Hartmaier R., Deaver K.E., Davis S., French J.D., Borre P.V., LaBarbera D.V., Tan A.C. (2018). Genetic Analysis of 779 Advanced Differentiated and Anaplastic Thyroid Cancers. Clin. Cancer Res..

[8] Cleere E.F., Prunty S., O’Neill J.P. (2024). Anaplastic thyroid cancer:Improved understanding of what remains a deadly disease. Surgeon.

[9] Capdevila J., Wirth L.J., Ernst T., Ponce Aix S., Lin C.C., Ramlau R., Butler M.O., Delord J.P., Gelderblom H., Ascierto P.A. (2020). PD-1 Blockade in Anaplastic Thyroid Carcinoma. J. Clin. Oncol..

[10] Baek H.W., Han E., Oh K.H. (2024). Exploring the Role of the KCNK1 Potassium Channel and Its Inhibition Using Quinidine in Treating Head and Neck Squamous Cell Carcinoma. Clin. Exp. Otorhinolaryngol..

[11] Subbiah V., Kreitman R.J., Wainberg Z.A., Cho J.Y., Schellens J.H.M., Soria J.C., Wen P.Y., Zielinski C., Cabanillas M.E., Urbanowitz G. (2018). Dabrafenib and Trametinib Treatment in Patients with Locally Advanced or Metastatic BRAF V600-Mutant Anaplastic Thyroid Cancer. J. Clin. Oncol..

[12] Smallridge R.C., Ain K.B., Asa S.L., Bible K.C., Brierley J.D., Burman K.D., Kebebew E., Lee N.Y., Nikiforov Y.E., Rosenthal M.S. (2012). American Thyroid Association guidelines for management of patients with anaplastic thyroid cancer. Thyroid.

[13] Hidalgo M., Amant F., Biankin A.V., Budinská E., Byrne A.T., Caldas C., Clarke R.B., de Jong S., Jonkers J., Mælandsmo G.M. (2014). Patient-derived xenograft models: An emerging platform for translational cancer research. Cancer Discov..

[14] Tentler J.J., Tan A.C., Weekes C.D., Jimeno A., Leong S., Pitts T.M., Arcaroli J.J., Messersmith W.A., Eckhardt S.G. (2012). Patient-derived tumour xenografts as models for oncology drug development. Nat. Rev. Clin. Oncol..

[15] Fior R., Póvoa V., Mendes R.V., Carvalho T., Gomes A., Figueiredo N., Ferreira M.G. (2017). Single-cell functional and chemosensitive profiling of combinatorial colorectal therapy in zebrafish xenografts. Proc. Natl. Acad. Sci. USA.

[16] White R.M., Sessa A., Burke C., Bowman T., LeBlanc J., Ceol C., Bourque C., Dovey M., Goessling W., Burns C.E. (2008). Transparent adult zebrafish as a tool for in vivo transplantation analysis. Cell Stem Cell.

[17] Rouhi P., Jensen L.D., Cao Z., Hosaka K., Länne T., Wahlberg E., Steffensen J.F., Cao Y. (2010). Hypoxia-induced metastasis model in embryonic zebrafish. Nat. Protoc..

[18] Astell K.R., Sieger D. (2020). Zebrafish In Vivo Models of Cancer and Metastasis. Cold Spring Harb. Perspect. Med..

[19] Zhang J., Yang C., Tang P., Chen J., Zhang D., Li Y., Yang G., Liu Y., Zhang Y., Wang Y. (2022). Discovery of 4-Hydroxyquinazoline Derivatives as Small Molecular BET/PARP1 Inhibitors That Induce Defective Homologous Recombination and Lead to Synthetic Lethality for Triple-Negative Breast Cancer Therapy. J. Med. Chem..

[20] Liu Y., Zhang G.Y., Li Y., Zhang Y.N., Zheng S.Z., Zhou Z.X., An S.J., Jin Y.H. (2013). Synthesis of novel amide derivatives with in vitro antiproliferative and cytotoxic activity. Heteroat. Chem..

[21] Yildiz I., Lee K.L., Chen K., Shukla S., Steinmetz N.F. (2013). Infusion of imaging and therapeutic molecules into the plant virus-based carrier cowpea mosaic virus: Cargo-loading and delivery. J. Control. Release.

[22] Hofmann M.C., Kunnimalaiyaan M., Wang J.R., Busaidy N.L., Sherman S.I., Lai S.Y., Zafereo M., Cabanillas M.E. (2022). Molecular mechanisms of resistance to kinase inhibitors and redifferentiation in thyroid cancers. Endocr. Relat. Cancer.

[23] Gild M.L., Bullock M., Tsang V., Clifton-Bligh R.J., Robinson B.G., Wirth L.J. (2023). Challenges and Strategies to Combat Resistance Mechanisms in Thyroid Cancer Therapeutics. Thyroid.

[24] Subbiah V., Kreitman R.J., Wainberg Z.A., Cho J.Y., Schellens J.H.M., Soria J.C., Wen P.Y., Zielinski C.C., Cabanillas M.E., Boran A. (2022). Dabrafenib plus trametinib in patients with BRAF V600E-mutant anaplastic thyroid cancer: Updated analysis from the phase II ROAR basket study. Ann. Oncol..

[25] Bible K.C. (2021). Immunotherapy in Anaplastic Thyroid Cancer: Much Yet to Be Learned. AACE Clin. Case Rep..

[26] Hatashima A., Archambeau B., Armbruster H., Xu M., Shah M., Konda B., Lott Limbach A., Sukrithan V. (2022). An Evaluation of Clinical Efficacy of Immune Checkpoint Inhibitors for Patients with Anaplastic Thyroid Carcinoma. Thyroid.

[27] Jeon M.J., Haugen B.R. (2022). Preclinical Models of Follicular Cell-Derived Thyroid Cancer: An Overview from Cancer Cell Lines to Mouse Models. Endocrinol. Metab..

